# Delivery of Nanoparticles across the Intestinal Epithelium via the Transferrin Transport Pathway

**DOI:** 10.3390/pharmaceutics11070298

**Published:** 2019-06-26

**Authors:** Jing M. Yong, Julia Mantaj, Yiyi Cheng, Driton Vllasaliu

**Affiliations:** School of Cancer and Pharmaceutical Sciences, Faculty of Life Sciences & Medicine, King’s College London, London SE1 9NH, UK

**Keywords:** Caco-2, intestinal absorption, nanomedicine, nanoparticle, oral delivery, transferrin

## Abstract

The aim of this study was to probe whether the transferrin (Tf) transport pathway can be exploited for intestinal delivery of nanoparticles. Tf was adsorbed on 100 nm model polystyrene nanoparticles (NP), followed by size characterisation of these systems. Cell uptake of Tf and Tf-adsorbed NP was investigated in intestinal epithelial Caco-2 cells cultured on multi-well plates and as differentiated polarised monolayers. Tf-NP demonstrated a remarkably higher cell uptake compared to unmodified NP in both non-polarised (5-fold) and polarised cell monolayers (16-fold difference). Application of soluble Tf significantly attenuated the uptake of Tf-NP. Notably, Tf-NP displayed remarkably higher rate (23-fold) of epithelial transport across Caco-2 monolayers compared to unmodified NP. This study therefore strongly suggests that the Tf transport pathway should be considered as a candidate biological transport route for orally-administered nanomedicines and drugs with poor oral bioavailability.

## 1. Introduction 

The oral drug administration route offers the ultimate patient convenience, preference and therefore adherence to drug therapy. However, with a few exceptions, oral administration is currently an option only for small drug molecules that show acceptable intestinal absorption. As a rapidly expanding class of drugs, biologics are presently predominantly given by injection. Significant research efforts over a number of decades have explored technologies to enable oral delivery of biologics, but progress has been relatively poor. Drug delivery strategies in this area mostly utilise absorption or permeation enhancers and focus on smaller biologics, such as glucagon-like peptide 1 (GLP-1) analogues [1]. However, safety concerns, including those related to many surfactants [2], have hindered the clinical translation of these approaches. Recent progress in this area seeks to utilise advances in materials, engineering and electronics, leading to swallowable “devices”, such as mucoadhesive patches [3] and the microneedle “robotic pill” [4]. 

The key challenge in the field of oral delivery of macromolecular biologics concerns the difficulty in overcoming the formidable intestinal epithelial barrier, rather than additional barriers such as the stomach acid and mucosal enzymes, which can be addressed via relatively established technologies. A key requirement for technologies enabling therapeutically-relevant oral delivery of biologics is safety. Rather than disrupting and increasing the permeability of the intestinal epithelium non-selectively (i.e., an effect that a classical permeation enhancer would display), it is desirable to engineer delivery systems that selectively permeate the intestinal mucosa. This can be achieved by targeting and hijacking the natural, physiological transport processes present in the intestinal epithelium. This approach usually requires a ligand or transport-enabling entity capable of intestinal translocation, which is linked (e.g., conjugated or fused) to the biotherapeutic. Alternatively, the ligand can be presented on the surface of drug carriers, particularly nanocarriers [5,6,7], which may also serve to protect the drug against mucosal enzymatic degradation or enable targeted delivery post absorption. 

Transferrin (Tf) has been explored as a potential ligand to enable drug targeting and delivery across biological barriers, particularly the blood-brain barrier (BBB) [8,9], because of its high expression in BBB endothelium [10]. The transferrin receptor (TfR) is expressed in the human gastrointestinal epithelial cells [11]. Furthermore, TfR is overexpressed in colon cancer (similarly to other types of cancer) [12] and the inflamed colon; it was detected in both basolateral and apical sides of enterocytes from the colon tissue biopsies of inflammatory bowel disease (IBD) patients and rats with colitis [13]. Therefore, TfR and TfR-mediated transcytosis could be exploited as a biological system for systemic delivery of biologics, in addition to its potential as a targeting receptor for local delivery in intestinal cancer or IBD. 

However, it must be noted that it is currently unclear whether TfR-mediated transcytosis offers opportunity for improving intestinal delivery of biologics in the context of systemic delivery, or local, targeted delivery to the intestinal diseased tissue. This is because of predominant distribution of TfR on the basolateral surface in polarised cells, which presents an obvious obstacle for receptor-mediated mucosal delivery [14]. There are however reports of TfR-mediated transcytosis being explored for oral delivery of biologics via a Tf recombinant fusion protein approach [15] or Tf conjugation to therapeutic macromolecule [16]—both these approaches have shown evidence and potential for the use of TfR-mediated transport as a strategy to enhance the intestinal absorption (i.e., in the apical-to-basolateral direction) of biologics. Importantly, a recent landmark study clearly showed a complete and enhanced apical-to-basolateral transcytosis of Tf-functionalised nanogranules in Caco-2 cells, with Tf modification upregulating the expression of trafficking-related (endocytosis and transcytosis) proteins [17].

This study probed the possibility of improving the intestinal epithelial delivery of nanoparticles (NP), as potentially useful carriers of biologics, by targeting TfR-mediated transcytosis. A fundamental study of this nature is imperative to assess the possibility that TfR-mediated transcytosis may be utilised to facilitate intestinal translocation of nanomedicines. This is important as, with the exception of two studies [17,18], TfR-mediated transcytosis has not been investigated for intestinal delivery of nanosystems. We show here that TfR targeting of NP significantly improves their uptake into intestinal epithelial cells (Caco-2), as well as translocation across cell monolayers serving as an in vitro intestinal model. 

## 2. Materials and Methods

### 2.1. Materials

Hank’s balanced salt solution (HBSS; with sodium bicarbonate, without phenol red), Dulbecco’s Modified Eagle’s Medium (DMEM) and all other reagents (including cell culture media supplements, antibiotic-antimycotic solution and foetal bovine serum), unless otherwise stated, were purchased from Sigma-Aldrich (Poole, UK). Fluorescently-labelled (“fluospheres”), carboxylate-modified polystyrene microspheres of 0.1 μm diameter (referred to as “NP”) and multiwell cell culture plates were purchased from Thermo Fisher Scientific (Waltham, MA, USA). Human holotransferrin (Tf) was purchased from LEE biosolutions (Maryland Heights, MO, USA), while fluorescently-labelled human Tf-CF^®^488A dye conjugate was purchased from Biotium (Fremont, CA, USA). Transwell permeable inserts of 12 mm diameter and polycarbonate filters and 0.4μm pore size were obtained from Corning (Corning, NY, USA). 

### 2.2. Adsorption of Tf to Nanoparticles

Tf-adsorbed nanoparticles (Tf-NP) were prepared based on a procedure that was previously reported [19]. A 1:2 Tf to NP mass ratio (1.1 mg/mL Tf and 2.2 mg/mL carboxylic-modified NPs) was used, with adsorption achieved by incubation in 50mM 2-(*N*-morpholino)ethanesulfonic acid (MES) buffer at pH 5.9 for 2 h at 20 °C. A previous study characterising Tf adsorption to latex NP reported the 1:1 mass ratio to produce full surface coverage on the same NP systems [19]. Given that we used a lower amount of Tf (below adsorption saturation), the presence of unadsorbed Tf was assumed to be very low and dialysis was not carried out. 

### 2.3. Nanoparticle Characterisation 

Average hydrodynamic size (dynamic light scattering, DLS), polydispersity and zeta potential of NP before and post Tf adsorption were measured by using a Malvern zetasizer (Malvern Instruments Ltd., Malvern, UK). Unmodified and Tf-NP were diluted in HBSS (biological buffer used in cell experiments). Measurements were conducted at a scattering angle θ = 173 and at a temperature of 25 °C. For DLS, each measurement was an average of 12 repetitions of 10 s each. Both DLS and zeta potential measurements were repeated three times. 

### 2.4. Cell Culture 

Caco-2 cells were cultured on 24-well plates for two days as undifferentiated system and Transwell inserts as differentiated monolayers, following plating at 10^5^ cells/cm^2^ using DMEM. Cells were cultured on inserts for at least 21 days prior to the experiments, with culture medium replaced every other day. For differentiated Caco-2 system, transepithelial electrical resistance (TEER) was measured periodically and before uptake and transport experiments to ensure integrity of the monolayer and tight junction formation (polarisation). Prior to cell uptake and transport experiments, both undifferentiated and differentiated Caco-2 cells were equilibrated in HBSS for 45 min to minimise the potential impact of media change on cell uptake and transport studies.

### 2.5. Cell Uptake of Tf 

Human Tf CF488A conjugate was applied to Caco-2 cells (cultured on 24-well plates) at 50 μg/mL and cells incubated at 37 °C for two hours. This was followed by removing the applied sample and repeated cell washing using HBSS. Triton X-100 (1% *v*/*v*) was applied to cells for 10 min to permeabilise and detach the cells. Permeabilised cells were then centrifuged and the supernatant harvested. Tf CF488A conjugate was quantified by measuring the fluorescence of supernatant using a Tecan Fluorescence Plate Reader (Tecan Trading AG, Männedorf, Switzerland) at 515 nm emission/490 nm excitation. Quantitation of samples was carried out via a calibration curve.

### 2.6. Cell Uptake of Tf-NP 

Tf-NPs (1:2 mass ratio) were applied to Caco-2 cells cultured on 24-well plates at 40 μg/mL. Cells were incubated with the samples at 37 °C for two hours, following washing with HBSS. Cells were then permeabilised via the application of Triton X-100 (1% *v*/*v* in HBSS). Cells were then harvested and transferred to 1 mL vials for centrifugation. Tf-NPs were quantified by measuring NP fluorescence following centrifugation of permeabilised cells and measurement of fluorescence of the supernatant using a Tecan fluorescence plate reader at 590 nm/645 nm (excitation/emission). 

#### 2.6.1. Competition Studies

For competition studies, cells were pre-treated with 10 μg/mL of Tf, shortly followed by application of Tf-NP. Cell uptake was examined as above. 

#### 2.6.2. Uptake in Differentiated Monolayers

In addition to examining uptake of Tf-NP in multiwell plate-grown Caco-2 cells, we also tested cell internalisation of these systems in differentiated Caco-2 cells (i.e., following culture on Transwell inserts). Only cell monolayers displaying TEER ≥ 500 Ωcm^2^ were used in the experiments (given the typical range observed in our work of 700–1400 Ωcm^2^). Application of Tf-NP and cell monolayer permeabilisation was conducted in the same manner as above. 

### 2.7. Transport of Tf-NP across Differentiated Caco-2 Monolayers 

Caco-2 cells were cultured as polarised monolayers on Transwell inserts as described above. Prior to the transport study, cells were equilibrated in HBSS. Tf-NP (1:2 ratio) were then applied to the apical side of Caco-2 cells at 40 μg/mL for two hours. Unmodified NPs were also applied at equivalent concentration. Cells were incubated with the samples at 37 °C for two hours, with periodic sampling of the basolateral solution every 20 min (this was replaced with fresh HBSS). Samples were transferred onto a black 96-well plate for NP fluorescence quantitation as above. 

### 2.8. Statistical Analysis 

Unpaired, unequal variance t test (or Welch t test) was performed for comparisons of two group means, while one-way analysis of variance (ANOVA) was utilised for comparison of three or more group means. *p* value of <0.05 was considered statistically significant. ***, ** and * indicate *p* < 0.001, *p* < 0.01 and *p* < 0.05, respectively, whereas “ns” denotes nonsignificant. Statistical analysis was conducted using GraphPad Prism^®^ Software. 

## 3. Results

**Figure 1:**
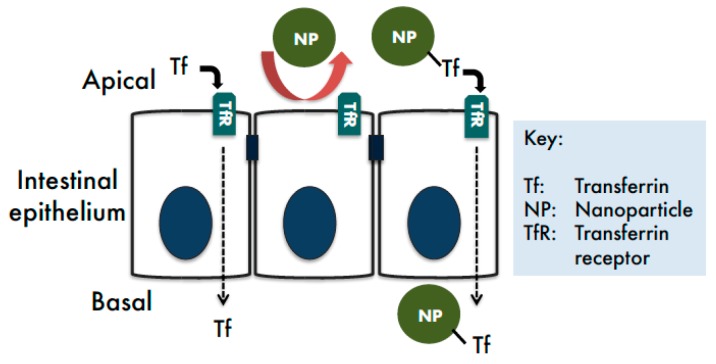
Transferrin transcytosis pathway as a potential route for intestinal delivery of nanomedicines.

This study examined whether TfR-mediated transcytosis may be utilised as a biological transport route to facilitate intestinal delivery of nanomedicines (Figure 1).

### 3.1. Nanoparticle Characterisation 

**Figure 2:**
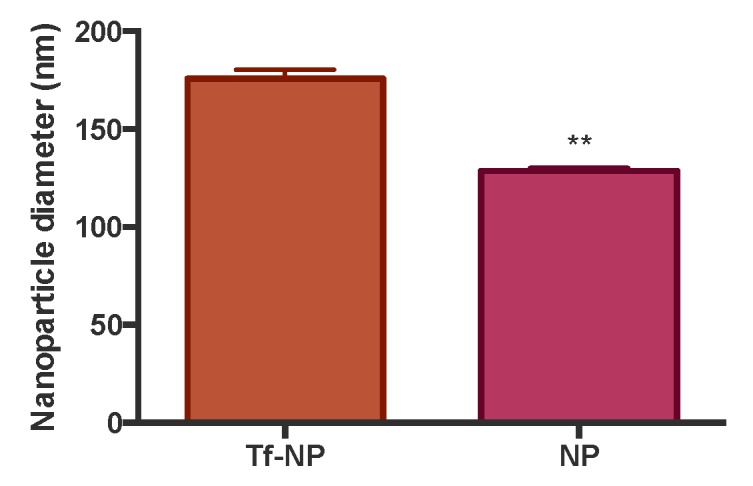
Hydrodynamic size of bare nanoparticles (NP) and transferrin-adsorbed systems (Tf-NP). Size was characterised by dynamic light scattering (DLS), with systems suspended in Hank’s Balanced Salt Solution (HBSS). Measurements were done at scattering angle θ = 173 and at a temperature of 25 °C. Data shown as mean ± SD. Each measurement was an average of 12 repetitions of 10 s each and repeated three times. ** denotes *p* < 0.01.

To assess the effect of physical adsorption of Tf to NP on their size, we conducted size characterisation of bare NP and Tf-NP. Data shown in Figure 2 highlight that adsorption of Tf on model polystyrene NP produced an increase in the hydrodynamic diameter of the NP (measured by DLS) from approximately 130 nm to 176 nm, indicating the formation of an adsorbed Tf surface layer of about 23 nm. 

In terms of surface charge, the zeta potential of unmodified NP was −35.7 (± 1.57), whereas for Tf-NP this amounted to −14.3 (± 1.03), resulting in a statistically significant reduction of negative surface charge post Tf adsorption (*p* = 0.0001). 

### 3.2. Cell Uptake of Tf

Uptake of Tf by multiwell-cultured (undifferentiated) Caco-2 cells following application at 50 μg/mL (at 37 °C for two hours) was 0.18 μg/well (24-well plate). 

### 3.3. Cell Uptake of Tf-NP 

**Figure 3:**
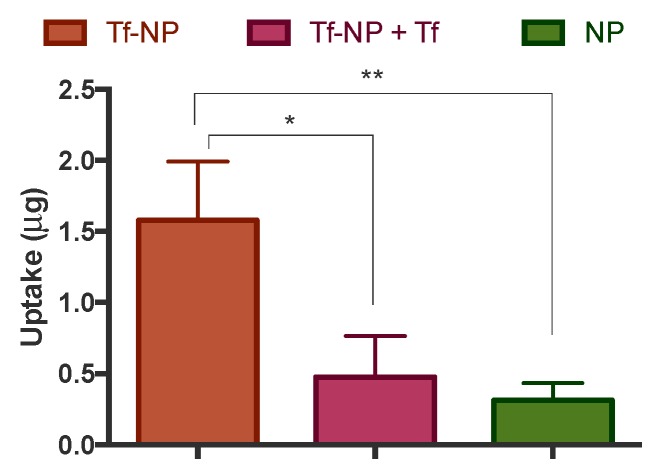
Uptake of transferrin-adsorbed nanoparticles (‘Tf-NP’) and bare nanoparticles (NP) by Caco-2 cells cultured on multiwell plates. Tf-NP were applied to cells at 40 μg/mL alone or just after application of 10 μg/mL soluble Tf (‘Tf-NP + Tf’). Cells were incubated with the samples at 37 °C for two hours. Cell internalisation was measured following permeabilization with Triton X-100 (1% *v*/*v* in Hank’s Balanced Salt Solution), centrifugation and measurement of nanoparticle fluorescence. Data shown as the mean ± SD (*n* = 3). ** and * denote *p* < 0.01 and *p* < 0.05, respectively.

**Figure 4:**
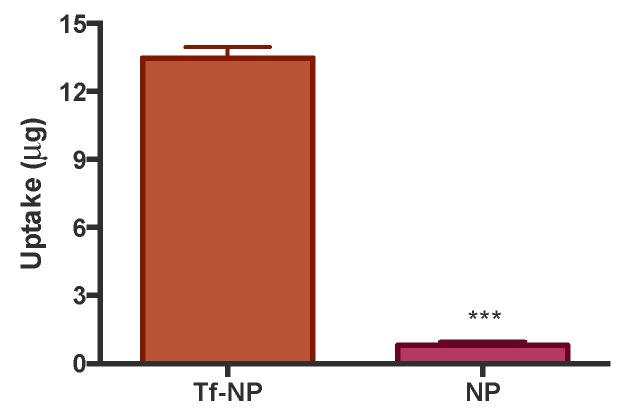
Uptake of transferrin-adsorbed nanoparticles (‘Tf-NP’) and bare nanoparticles (NP) by Caco-2 cells cultured as differentiated monolayers. Tf-NP were applied to the apical side of Caco-2 monolayers at 40 μg/mL in Hank’s Balanced Salt Solution (HBSS) as biological buffer. Cells were incubated with the samples at 37 °C for two hours. Cells were permeabilised via the application of 100 μL of Triton X-100 (1% *v*/*v* in HBSS). Nanoparticles were quantified by fluorescence. Data presented as the mean ± SD (*n* = 3). *** denotes *p* < 0.001

The internalisation of Tf-NP by intestinal Caco-2 cells was tested under different conditions in non-polarised, multiwell-cultured cells (Figure 3), prior to subsequent examination in differentiated cell monolayers (Figure 4). Considering the multiwell-cultured cells, Figure 2 shows the internalisation of Tf-NP after application alone, or following treatment with soluble Tf (‘+Tf’). The figure also depicts the uptake of bare NP. The data highlight more than five-fold higher cell uptake of Tf-NP compared to bare NP. Importantly, following cell treatment of Tf-NP in conjunction with excess free Tf, cell internalisation of the former was attenuated by more than three-fold. 

### 3.4. NP-Tf Uptake in, and Transport across, Differentiated Monolayers 

Cell uptake of Tf-NP in differentiated Caco-2 monolayers is depicted in Figure 4. The data demonstrate more than 16-fold higher cell uptake of Tf-NP compared to bare NP. 

**Figure 5:**
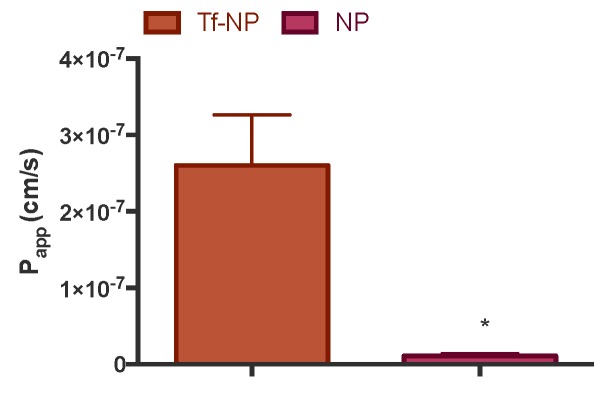
Transport of transferrin-adsorbed nanoparticles (‘Tf-NP’) and bare nanoparticles (NP) across differentiated Caco-2 cells (cultured on inserts for three weeks). Tf-NP were applied to the apical side of Caco-2 monolayers at 40 μg/mL in Hank’s Balanced Salt Solution (HBSS) as biological buffer. Cells were incubated with the samples at 37 °C for two hours, during which sampling from the basolateral compartment was carried out periodically. Data presented as the mean ± SD (*n* = 3). * denotes *p* < 0.05.

Following the demonstration of Tf-NP uptake by intestinal Caco-2 cells, the final set of experiments looked into the transepithelial transport. In this regard, Figure 5 shows that Tf-NP traversed differentiated Caco-2 monolayers remarkably more efficiently; specifically a 23-fold higher rate of NP translocation was observed with Tf-NP compared to bare counterparts. 

## 4. Discussion 

The intestinal mucosa acts as a selective barrier to systemic absorption of material present in the lumen. Large and complex biologics are usually not capable of crossing the intestinal epithelium, therefore displaying very low bioavailabilities following oral administration. One way to improve the oral bioavailability of such drugs is to enhance epithelial absorption by targeting the transcytosis pathways as physiological routes of transport in the intestinal epithelium. This strategy has been made possible by advances in biotechnology and nanotechnology, which have facilitated the production of fusion proteins and drug nanocarriers, respectively. We and other groups have demonstrated that a number of physiological transcytosis processes functioning in the intestinal epithelium can potentially be used to shuttle material (nanoparticles and macromolecules) across the intestinal epithelium, including the transport system for IgG [20,21], neurotensin [22] and albumin [23,24]. This study provides further progress in this arena, by demonstrating that the biological machinery for Tf transport can also be considered as a potential pathway for intestinal delivery of nanomedicines. 

Tf is an abundant human plasma glycoprotein responsible for transport of iron, binding to TfR when bound to iron, activating receptor mediated endocytosis [25]. As tumour cells generally overexpress the TfR because of high demand for iron, Tf-mediated drug delivery strategy has been predominantly used for its potential to target cancer cells, usually as a ligand to guide NP to these cells. 

Transferrin receptor (TfR)-mediated endocytosis and transcytosis in intestinal Caco-2 cells was previously investigated using Tf-fusion proteins. It was shown that apically-endocytosed Tf in Caco-2 cells is transported to a Rab11-positive compartment, which may control its release to follow either slow recycling to apical membrane or progress to transcytotic compartments [26]. Shah and Shen studied the transport of Tf-conjugated insulin across Caco-2 cell monolayers and reported that transport of the complex, which was mediated via the TfR, was increased by 5- to 15-fold compared to free insulin [16]. In another study, Tf recombinant fusion protein approach was explored for oral delivery for human growth hormone (hGH) [15]. Fusion proteins of 100 kDa, which retained bioactivity of both hGH and Tf, were tested in hGH-deficient hypophysectomised rats for in vivo biological activity. It was shown that one of the tested fusion proteins—containing a helical linker as spacer between hGH and Tf domain—induced a statistically significant weight gain after oral dosing.

We utilised fluorescently-labelled model polystyrene NP systems and achieved ligand (Tf) presentation on NP via physical adsorption. This produced Tf-NP of approximately 180 nm (Figure 2), which fall within the optimal size range for intestinal epithelial transport [6,20,27,28]. Furthermore, it was previously shown that following presentation as coating on the surface of NP via physical adsorption, Tf retains the ability to target and interact with TfR [29]. 

We first probed the internalisation of Tf by Caco-2 cells, demonstrating that the extent of Tf uptake by Caco-2 cells (0.18 μg/1.9 cm^2^) is likely to be comparable with a previous study [16] showing that cell internalization of insulin-Tf conjugate was around 1.9 pmoles per 3.5 cm^2^ monolayer (Caco-2), equivalent to around 0.15 μg (the final insulin:Tf ratio in the conjugate was 3:1). 

Cell internalisation of Tf-NP was tested both in multiwell-cultured cells and differentiated cell monolayers. This was done to determine any potential difference in cell uptake capacity between undifferentiated and differentiated cells (cultured on inserts), given the difference in surface area available for uptake and potential differential receptor expression between undifferentiated and differentiated cells. In undifferentiated cells (multiwell culture) cell uptake of Tf-NP was over five-fold higher relative to bare NP. Furthermore, excess free Tf reduced cell uptake of Tf-NP by more than three-fold (Figure 3). With regards to cell uptake of Tf-NP in differentiated cells, data revealed more than 16-fold higher cell uptake of Tf-NP compared to bare NP (Figure 4), with the latter displaying a similar uptake level to that previously reported by us (around 2 μg here versus 5 μg in previously published work [30]), confirming the pattern observed in undifferentiated cells. The notably larger magnitude of effect (enhanced cell uptake following Tf presentation on NP) may be related to differential TfR expression in undifferentiated *versus* differentiated cells. The implications of these findings are important, pointing to significant enhancement of cell uptake of NP following presentation of Tf on their surface.

It is noted that the definitive biological pathway of Tf-NP uptake in Caco-2 cells cannot be ascertained from this work. However, suppression of internalisation by excess free Tf does point to a TfR-dependent uptake, similarly to the study by Shah and Shen [16] who demonstrated that the binding and uptake of the radiolabelled insulin-Tf conjugate in Caco-2 cells were inhibited by 70% and 80%, respectively, with excess unlabeled Tf. 

We finally considered the transport of Tf-NP across differentiated Caco-2 monolayers. The data concerning this showed remarkably higher (23-fold) transport of Tf-NP across cells compared to bare NP (Figure 5). It must be pointed out that this remarkable enhancement in the permeation of NP across intestinal epithelium in vitro is much higher than that previously seen with other transcytosis receptor systems exploited in a similar manner (i.e., for intestinal NP delivery). This includes the neonatal Fc receptor (FcRn), whereby the uptake into Caco-2 cells of polyethylene glycol-poly(lactic-*co*-glycolic acid) (PEG-PLGA) NP modified with Fc was improved (compared to non Fc-modified NP) by a maximum three-fold [20]. Similarly, a different study showed that the translocation of 63 nm Fc-targeted poly(lactic acid)-*b*-poly(ethylene glycol) (PLA-PEG) across Caco-2 monolayers was increased by approximately a factor of two (compared to non-modified NP) [21]. 

Overall, this work highlights that the TfR transport system shows potential as a pathway for cell entry and transepithelial permeation of NP in the intestinal epithelium. Therefore, this system should be given consideration as a potential pathway for intestinal delivery of nanomedicines intended for systemic effect or local action. The latter is particularly applicable to colon cancer and IBD, given TfR overexpression in the intestinal mucosa in these diseases [12,13]. Therefore, TfR-mediated delivery should be probed further for its potential usefulness in the area of oral or intestinal delivery of nanomedicines.
